# Perimenopause as a potential window of cardiometabolic remodeling in heart failure with preserved ejection fraction: a missed opportunity for prevention

**DOI:** 10.3389/fcvm.2026.1948320

**Published:** 2026-09-03

**Authors:** Faith Adedayo Adejumo, Oluwayimika Oluwatobi Obielodan, Fiyinfoluwa Adetoye, Nicholas Aderinto

**Affiliations:** 1 Department of Medicine and Surgery, Bowen University, Iwo, Nigeria; 2 Department of Medicine and Surgery, Ladoke Akintola University of Technology, Ogbomoso, Nigeria

**Keywords:** cardiometabolic remodeling, coronary microvascular dysfunction (CMD), heart failure with preserved ejection fraction, menopausal transistion, perimenopause

## Abstract

Heart failure with preserved ejection fraction (HFpEF) disproportionately affects women and has emerged as one of the most prevalent forms of heart failure worldwide. Although the marked increase in HFpEF incidence after menopause is well recognized, the biological events that initiate disease development remain poorly defined. Most mechanistic and clinical investigations have focused on postmenopausal women or established HFpEF, leaving the menopausal transition comparatively underexplored despite growing evidence that it represents a period of accelerated cardiometabolic change. This review synthesizes current evidence on reproductive aging, cardiometabolic remodeling, and the pathophysiological mechanisms underlying HFpEF to examine whether perimenopause represents a critical window in the development of the female HFpEF phenotype. Evidence from longitudinal cohorts and mechanistic studies indicates that visceral and epicardial adiposity, insulin resistance, adverse lipid remodeling, systemic inflammation, endothelial dysfunction, coronary microvascular impairment, arterial stiffening, and early cardiac remodeling emerge or accelerate during the menopausal transition. These biological alterations closely parallel established mechanisms implicated in HFpEF pathogenesis. Building on prior clinical and experimental evidence identifying the menopausal transition as a period of cardiovascular remodeling and increasing cardiovascular risk, we develop a temporal framework for female HFpEF. This framework considers whether, in susceptible women, HFpEF-relevant biological changes may begin to accumulate during perimenopause, become progressively amplified after menopause, and ultimately contribute to clinically manifest disease. We further examine how reliance on binary reproductive classifications, recruitment of predominantly postmenopausal cohorts, and the limited integration of reproductive staging into cardiovascular research may have contributed to this early phase of disease remaining largely unrecognized. Reframing perimenopause as a critical period of cardiovascular remodeling shifts attention from treating established HFpEF to identifying opportunities for earlier prevention. Prospective longitudinal studies incorporating standardized reproductive staging, comprehensive cardiovascular phenotyping, and biomarker profiling are now needed to determine whether the menopausal transition represents the earliest identifiable window for preventing HFpEF in women.

## 1 Introduction

### 1.1 HFpEF as a female-predominant syndrome

Heart failure with preserved ejection fraction (HFpEF) now accounts for approximately 50% of all heart failure cases and is characterized by a marked female predominance (1, 2). Across contemporary HFpEF cohorts, heart failure registries, and randomized clinical trials, women consistently comprise the majority of affected patients, a pattern that contrasts sharply with heart failure with reduced ejection fraction, where men predominate (3–5).

This sex disparity carries important clinical consequences, as women with HFpEF experience substantial symptom burden, recurrent hospitalization, impaired functional capacity, and diminished quality of life despite advances in contemporary heart failure management (6). Although this epidemiological pattern is well established, its biological basis remains incompletely understood. Differences in ventricular remodeling, vascular aging, adipose tissue biology, and cardiometabolic adaptation have each been proposed to contribute to the female predominance of HFpEF (7, 8), yet none fully explains why women are disproportionately affected. Increasingly, HFpEF is recognized as a chronic, progressive syndrome that develops through the cumulative interaction of metabolic, vascular, inflammatory, and myocardial remodeling over many years before clinical heart failure becomes manifest (9).

Consequently, the central unanswered question is not whether biological sex influences HFpEF risk but rather when, across the female life course, the trajectory toward this sex-specific phenotype begins. Identifying the earliest stage of disease development has important implications not only for understanding HFpEF pathobiology but also for redefining opportunities for prevention before irreversible cardiac remodeling has become established.

### 1.2 The current HFpEF timeline

Most current understanding of female HFpEF is largely organized around a postmenopausal framework (3, 7). The marked increase in cardiovascular risk observed after the final menstrual period has focused attention on established menopause as the principal period during which female-specific mechanisms contribute to HFpEF development (10, 11). Consequently, mechanistic investigations, epidemiological studies, cardiovascular imaging cohorts, and emerging preventive strategies have overwhelmingly examined women after reproductive aging has been completed, implicitly positioning menopause as the point at which female-specific cardiovascular risk becomes most clinically apparent.

This framework has substantially advanced understanding of established HFpEF. Oestrogen deficiency has been linked to endothelial dysfunction, adverse adipose tissue redistribution, systemic inflammation, coronary microvascular dysfunction, myocardial fibrosis, arterial stiffening, and impaired ventricular relaxation (12). These processes are now recognized as central to HFpEF pathophysiology. Given that these processes develop progressively over years, it seems unlikely that the underlying pathological cascade is initiated only after ovarian function has ceased completely.

An important consequence of this postmenopause-centered perspective is that the architecture of cardiovascular research has likewise become centered on established menopause. Women are commonly classified as either premenopausal or postmenopausal, whereas the intervening perimenopausal transition has received comparatively less attention as a distinct stage of cardiovascular investigation despite evidence that important cardiometabolic and vascular remodeling occurs during this period (13, 14). As a result, current research frameworks may be exceptionally well suited to characterizing established disease while being considerably less effective at identifying the period during which its biological substrate is first assembled.

### 1.3 The missing decade

Perimenopause represents the transitional period during which ovarian function becomes increasingly irregular before the final menstrual period (15). Rather than constituting a simple decline in ovarian hormone production, this stage is characterized by marked endocrine instability, with fluctuating concentrations of estrogen and progesterone occurring alongside progressive changes in metabolic and vascular physiology (15). This transition represents one of the most dynamic periods of cardiometabolic remodeling across the female lifespan.

Longitudinal studies have consistently demonstrated that many cardiovascular risk factors traditionally attributed to postmenopause, including visceral adiposity, insulin resistance, adverse lipid remodeling, systemic inflammation, endothelial dysfunction, and increasing arterial stiffness, begin to accelerate during the menopausal transition itself (10). Importantly, these biological alterations closely parallel the mechanisms increasingly recognized as fundamental to contemporary models of HFpEF pathogenesis (16). Because these biological alterations are progressive rather than abrupt and begin during the menopausal transition, they raise the possibility that biological changes relevant to future HFpEF may accumulate before menopause is complete. Despite this convergence, relatively little cardiovascular research has examined perimenopause as a distinct stage of disease development. Instead, it has generally been regarded as a transitional interval preceding menopause rather than as a biologically active period during which the substrate for future HFpEF may first emerge.

This disconnect represents a potentially important conceptual gap. If cardiometabolic and cardiovascular remodeling accelerate during perimenopause, then a research framework centered predominantly on postmenopausal women may be observing the consequences of biological remodeling rather than its earliest origins. Under this interpretation, the years immediately preceding menopause may represent not merely a transition between reproductive stages but a previously underrecognized window during which susceptibility to future HFpEF may increase.

### 1.4 Central thesis

The concept that the menopausal transition represents an important period of cardiovascular remodeling and increasing cardiovascular risk predates this present review. Longitudinal clinical research has demonstrated adverse cardiometabolic and vascular changes during the menopausal transition, and the American Heart Association has recognized this period as a window of accelerating cardiovascular risk and an opportunity for earlier prevention (13). Experimental studies have further demonstrated cardiac molecular remodeling during the perimenopausal period in models of accelerated ovarian failure (16). Building on these established observations, this review examines their implications for the temporal development of female-predominant HFpEF.

Within this framework, the available evidence is organized into a temporal sequence in which perimenopause represents a potential initiation or early remodeling phase, postmenopause an amplification phase, and clinical HFpEF a late manifestation of accumulated cardiometabolic, vascular, and myocardial abnormalities in susceptible women. We use the term “temporal origin framework” to describe this HFpEF-specific synthesis of existing evidence, rather than to claim novelty for the underlying concept that cardiovascular risk and remodeling evolve during the menopausal transition. The framework does not imply that perimenopause independently causes HFpEF; rather, it places perimenopausal remodeling within a multifactorial trajectory in which aging, metabolic risk, vascular dysfunction, and other individual factors may interact to influence subsequent disease development.

This framework generates several testable predictions. First, greater cardiometabolic deterioration during perimenopause should be associated with a greater subsequent burden of structural and functional cardiac abnormalities. Second, vascular and coronary microvascular abnormalities emerging during the transition should predict later diastolic dysfunction and incident HFpEF. Third, the magnitude and timing of perimenopausal remodeling should provide prognostic information beyond conventional cardiovascular risk factors. Finally, if perimenopause represents an early remodeling window, interventions initiated during this period may have greater potential to attenuate subsequent HFpEF-related remodeling than those initiated after substantial structural disease has developed.

Building on this perspective, we examine the biological distinctiveness of perimenopause, synthesize evidence for accelerated cardiometabolic remodeling during the menopausal transition, explore the mechanistic links between these changes and HFpEF biology, and integrate human and preclinical evidence to evaluate whether perimenopause should be incorporated into the temporal model of female HFpEF. Finally, we consider the implications of this framework for cardiovascular research, early risk stratification, preventive intervention, and future longitudinal investigation aimed at determining when the biological trajectory toward female HFpEF may begin.

## 2 Methods

This narrative review synthesizes current evidence on reproductive aging, cardiometabolic remodeling, and the pathophysiological mechanisms underlying heart failure with preserved ejection fraction (HFpEF) to examine whether perimenopause represents a critical window of cardiovascular remodeling relevant to subsequent HFpEF development. A literature search was conducted across PubMed, Google Scholar, Cochrane and Web of Science using combinations of the following search terms: “*perimenopause,” “menopausal transition,” “reproductive aging,” “heart failure with preserved ejection fraction,” “HFpEF,” “cardiometabolic remodeling,” “endothelial dysfunction,” “coronary microvascular dysfunction,” “arterial stiffness,” “myocardial fibrosis,” “diastolic dysfunction,”* and “*women's cardiovascular health.”* Additional relevant publications were identified through manual screening of reference lists from eligible articles, major cardiovascular guidelines, and consensus statements.

Peer-reviewed original research articles, systematic reviews, meta-analyses, clinical practice guidelines, consensus statements, and landmark studies relevant to perimenopause, cardiovascular remodeling, and HFpEF were considered for inclusion. Priority was given to recent, high-quality evidence, particularly longitudinal cohort studies, mechanistic investigations, systematic reviews, meta-analyses, and contemporary clinical guidelines. Earlier publications were included where they represented landmark studies or established foundational concepts central to reproductive aging, HFpEF pathophysiology, or cardiovascular risk in women.

Two authors (FAA and NA) independently selected sources for inclusion; disagreements were resolved through discussion and consensus among the authorship group. No strict date restrictions were applied; however, priority was given to recent, high-quality evidence.

The identified evidence was narratively synthesized into four thematic domains: (1) perimenopause as a distinct biological stage; (2) cardiometabolic remodeling during the menopausal transition; (3) mechanistic links between perimenopausal remodeling and the HFpEF phenotype; and (4) the implications of these findings for the temporal origin framework.

## 3 Perimenopause as a distinct biological state

### 3.1 Defining perimenopause

According to the Stages of Reproductive Aging Workshop (STRAW+10), the menopausal transition comprises an early stage characterized by persistent variability in menstrual cycle length and a late stage marked by prolonged episodes of amenorrhea before the FMP, after which menopause is retrospectively established following 12 months of amenorrhea (15). Although the duration of this transition varies between individuals, it typically extends over several years and represents a dynamic stage of reproductive aging rather than an abrupt endocrine event.

The menopausal transition is therefore best regarded as a continuum encompassing progressive alterations in ovarian function before the establishment of the stable endocrine environment of postmenopause (10, 17). This distinction provides an important biological framework for understanding the physiological adaptations that accompany reproductive aging.

### 3.2 Hormonal instability rather than hormonal deficiency

The defining endocrine feature of perimenopause is not sustained estrogen deficiency but marked hormonal instability. As ovarian reserve declines, ovulatory cycles become increasingly irregular, resulting in substantial fluctuations in circulating estrogen and progesterone rather than a simple, linear decline (13). Women going through perimenopause are therefore exposed to repeated, and often unpredictable, hormonal oscillation well before the relatively stable hypoestrogenic state of postmenopause is reached.

The physiological implications of this instability extend well beyond the reproductive axis itself. Ovarian hormones exert regulatory influence over vascular tone, adipose tissue distribution, glucose and lipid metabolism, and inflammatory signaling (12, 14). During perimenopause, these systems adapt continuously to an evolving endocrine environment rather than to a fixed state of hormone deficiency. In this sense, the menopausal transition constitutes a period of active physiological remodeling, not merely a passive antecedent to hormonal decline, a distinction that recurs throughout this review and underlies its central argument.

This also positions perimenopause as endocrinologically distinct from both the reproductive years that precede it and the postmenopausal years that follow. Reproductive life is characterized by relatively regular and largely predictable cyclical variation (18), while postmenopause is characterized by persistently low and comparatively stable ovarian hormone concentrations (10). Perimenopause occupies neither condition; rather, it is defined by a degree of endocrine unpredictability that has no equivalent at either end of the transition, a distinction with direct consequences for how its cardiovascular biology should be studied and interpreted (10).

### 3.3 Implications for HFpEF research

This endocrine distinction carries a direct consequence for how HFpEF-relevant biology should be studied in midlife women. Because postmenopausal hormone levels remain relatively stable over time, a single measurement taken at any point after the final menstrual period is likely to reflect a woman's postmenopausal status fairly accurately, regardless of exactly when that measurement is taken. Perimenopause admits no equivalent assumption, as a measurement taken at one point captures only a moment within a continuously shifting hormonal trajectory and reveals little about where within that trajectory a given woman is positioned. For instance, two women both classified as perimenopausal may differ substantially in circulating hormone concentrations and metabolic profile depending on whether they are early or late within the transition (13), a degree of biological heterogeneity that is substantially greater than that observed once the relatively stable endocrine milieu of postmenopause has been established.

Cardiovascular research designs that treat perimenopausal women as a single, undifferentiated group, or that substitute postmenopausal data for perimenopausal characterization, are consequently poorly positioned to detect biology specific to this transition, even when perimenopausal participants are nominally included in HFpEF-relevant cohorts. Capturing an actively remodeling state requires designs capable of tracking change within the same woman over time, rather than the single time point, premenopausal-versus-postmenopausal comparisons that have dominated the HFpEF literature to date.

Recognizing both the biological distinctiveness of perimenopause and the methodological demands this distinctiveness imposes provides the necessary foundation for the evidence reviewed in the following section, which examines the specific cardiometabolic changes documented across the menopausal transition and their relevance to later HFpEF development.

## 4 Cardiometabolic, vascular, and myocardial remodeling during perimenopause: relevance to HFpEF

The menopausal transition is accompanied by coordinated changes in adiposity, metabolism, inflammation, vascular function, and cardiac structure. Rather than occurring as isolated abnormalities, these changes interact across biological levels and overlap with established pathways implicated in HFpEF. However, the strength of evidence varies across pathways: some changes are directly demonstrated during perimenopause, whereas others remain mechanistically plausible without direct evidence of progression to HFpEF. Integrating these findings provides a framework for considering perimenopause as a potential early remodeling window in the trajectory toward female-predominant HFpEF.

### 4.1 Adiposity, insulin resistance, and dyslipidemia

The most consistent metabolic changes during the menopausal transition involve redistribution of adipose tissue, particularly expansion of visceral and epicardial fat. Longitudinal SWAN data indicate that increases in adiposity and declines in lean mass accelerate during the menopausal transition rather than reflecting chronological aging alone (10, 19). Greater abdominal fat accumulation is also associated with deterioration in insulin sensitivity, supporting visceral adiposity as an important mediator of perimenopausal metabolic dysfunction (20).

Epicardial adipose tissue (EAT) similarly increases across reproductive aging, with imaging studies demonstrating greater EAT volume in late perimenopausal and postmenopausal women compared with premenopausal women, independent of chronological age and overall adiposity (21). This is relevant to HFpEF because EAT is associated with adverse cardiometabolic profiles, coronary microvascular dysfunction, diastolic dysfunction, and HFpEF (22–24). Thus, perimenopausal fat redistribution may contribute to the inflammatory, metabolic, and vascular milieu associated with HFpEF susceptibility.

Adverse lipid remodeling also accelerates around the menopausal transition, with increases in total cholesterol, LDL cholesterol, and apolipoprotein B concentrated around the final menstrual period (25). In SWAN, menopause-related increases in LDL cholesterol were associated with greater subsequent carotid plaque burden, suggesting downstream vascular consequences of these lipid changes (26). Although dyslipidemia is less directly linked to HFpEF than visceral adiposity, inflammation, or vascular dysfunction, it forms part of the broader cardiometabolic remodeling accompanying reproductive aging.

### 4.2 Inflammation, endothelial dysfunction, and vascular remodeling

Low-grade systemic inflammation increases during the menopausal transition, with longitudinal studies demonstrating rising hs-CRP and interleukin-6 as women approach the final menstrual period (13). This inflammatory shift may partly reflect changes in adipose tissue biology and altered estrogen-mediated regulation of inflammatory pathways (27). Inflammation subsequently interacts with endothelial dysfunction, vascular remodeling, and myocardial fibrosis, processes central to HFpEF pathophysiology.

Endothelial dysfunction, characterized by reduced nitric oxide (NO) bioavailability and impaired vasodilatory reserve, provides an important mechanistic link between systemic remodeling and HFpEF (1, 9, 28). Disruption of the NO–cGMP–protein kinase G (PKG) pathway can promote vascular dysfunction, myocardial stiffening, and adverse remodeling (29). Importantly, endothelial impairment is detectable before menopause is complete. In healthy women classified according to STRAW reproductive staging, brachial artery flow-mediated dilation was approximately 17% lower in early perimenopause and 35% lower in late perimenopause than in premenopausal controls, despite the absence of overt cardiovascular disease (30). Clinical studies in HFpEF further demonstrate impaired endothelial function and associations with exercise intolerance and disease severity (9, 28).

Structural vascular remodeling also accompanies the transition. In the SWAN Heart Study, greater accumulation of abdominal visceral adipose tissue during menopause transition was associated with greater carotid intima-media thickness in women without overt cardiovascular disease (31). Arterial stiffness is relevant to HFpEF because increased arterial load can impair ventricular relaxation and contribute to abnormal ventricular-arterial coupling (32, 33). Moreover, studies in women with HFpEF have linked arterial stiffness with coronary microvascular dysfunction, suggesting interconnected vascular and microvascular pathways (34). Together, these findings indicate that vascular abnormalities emerge during perimenopause and overlap with clinically relevant HFpEF mechanisms, although direct evidence linking perimenopausal vascular remodeling to incident HFpEF remains unavailable.

### 4.3 Coronary microvascular dysfunction

Coronary microvascular dysfunction (CMD) is an important component of HFpEF pathophysiology and may be particularly relevant to the female phenotype (35). For instance, a 2024 meta-analysis estimated CMD prevalence at more than 70% among patients with HFpEF (36), highlighting its high burden within the syndrome. In addition, another study demonstrated that CMD was associated with higher left atrial volume index, elevated E/e′ ratio, and an increased risk of death or heart failure hospitalization, supporting its role as a clinically meaningful determinant of disease severity and prognosis (37). These findings support CMD as a clinically meaningful component of the HFpEF phenotype rather than simply an incidental vascular abnormality.

Although no longitudinal study has directly examined coronary microvascular function across perimenopause, the convergence of these established drivers within this biological window raises the possibility that coronary microvascular remodeling may begin before the onset of established menopause rather than only after ovarian function has ceased.

### 4.4 Myocardial remodeling, fibrosis, and diastolic dysfunction

The metabolic, inflammatory, and vascular abnormalities described above may ultimately converge on myocardial remodeling. Myocardial fibrosis, impaired ventricular compliance, and concentric remodeling are established features of HFpEF and are promoted by interacting inflammatory, microvascular, endothelial, and metabolic pathways (8, 9, 38–40). Reduced NO bioavailability, systemic inflammation, CMD, and vascular dysfunction can therefore provide a biological environment conducive to myocardial stiffening and fibrotic remodeling (8). However, direct demonstration that myocardial fibrosis begins during perimenopause remains lacking.

Human longitudinal data nevertheless indicate that structural cardiac remodeling accompanies reproductive aging. In the CARDIA cohort, progression through natural menopause was associated with increased relative wall thickness and left ventricular mass-to-volume ratio (41). These associations were substantially attenuated after adjustment for cardiometabolic risk factors, suggesting that metabolic remodeling contributes importantly to the structural cardiac phenotype emerging across reproductive aging.

Diastolic dysfunction represents the functional abnormality most directly relevant to HFpEF, yet evidence across the perimenopausal transition remains limited. No longitudinal study has comprehensively assessed diastolic function across this transition using standardized reproductive staging. Thus, while endothelial dysfunction, CMD, arterial remodeling, and adverse left ventricular remodeling are demonstrable or mechanistically plausible during reproductive aging, whether their cumulative effects produce subclinical diastolic dysfunction during perimenopause remains unknown.

### 4.5 Insights from preclinical models of reproductive aging

Preclinical models provide complementary evidence by allowing cardiovascular changes associated with reproductive aging to be examined under controlled conditions. Traditional rodent models have relied heavily on ovariectomy, which produces abrupt ovarian hormone withdrawal and therefore does not reproduce the gradual ovarian follicle depletion characteristic of human reproductive aging (42). The 4-vinylcyclohexene diepoxide (VCD) model induces selective depletion of small ovarian follicles through accelerated atresia and provides a model of progressive ovarian failure that more closely approximates aspects of reproductive aging than surgical ovariectomy (42). However, VCD remains an accelerated experimental model and does not fully reproduce human perimenopause.

Studies using VCD models have identified cardiovascular changes at molecular, myocardial, vascular, and systemic levels. Left ventricular systolic function may remain preserved despite alterations in myocardial myofilament function, including changes in actomyosin MgATPase activity, Akt activation, and myofilament protein phosphorylation (43). Other experimental work has demonstrated increased BNP expression, cardiac hypertrophy and fibrosis, and greater susceptibility to cardiovascular injury following VCD-induced reproductive aging (44). VCD models have also demonstrated impaired baroreflex sensitivity, increased sympathetic vascular activity, impaired endothelium-dependent relaxation, and alterations in inflammatory and oxidative stress pathways accompanied by reductions in nitric oxide and glutathione (45–47).

Several of these findings overlap with established biological features of HFpEF, including endothelial and vascular dysfunction, inflammation, oxidative stress, myocardial fibrosis, hypertrophy, and altered myocardial signaling (48). Preclinical evidence therefore strengthens the biological plausibility that reproductive aging can influence multiple interconnected cardiovascular pathways relevant to HFpEF. Nevertheless, VCD-induced ovarian failure occurs over a substantially compressed timeframe, and the young animals commonly studied do not reproduce the simultaneous effects of chronological aging that accompany human menopause (49, 50). Moreover, these models demonstrate HFpEF-relevant mechanisms rather than the clinical HFpEF syndrome itself. Preclinical findings should therefore be interpreted as mechanistic support rather than evidence that perimenopause causes HFpEF in humans.

### 4.6 Temporal framework: from perimenopausal remodeling to clinical HFpEF

HFpEF is clinically recognized only after symptoms, elevated filling pressures, and objective cardiac abnormalities have developed, but its underlying structural and functional changes evolve over a much longer preclinical period (38, 39, 51, 52). HFpEF is increasingly understood as a heterogeneous syndrome arising from prolonged interactions among metabolic, inflammatory, vascular, and myocardial abnormalities rather than as a condition that begins abruptly at the time of clinical diagnosis. Within this context, the convergence of HFpEF-relevant biological changes during the menopausal transition raises the possibility that the trajectory of female-predominant HFpEF may begin before established menopause.

#### 4.6.1 Perimenopause as the initiation phase

**Figure 1:**
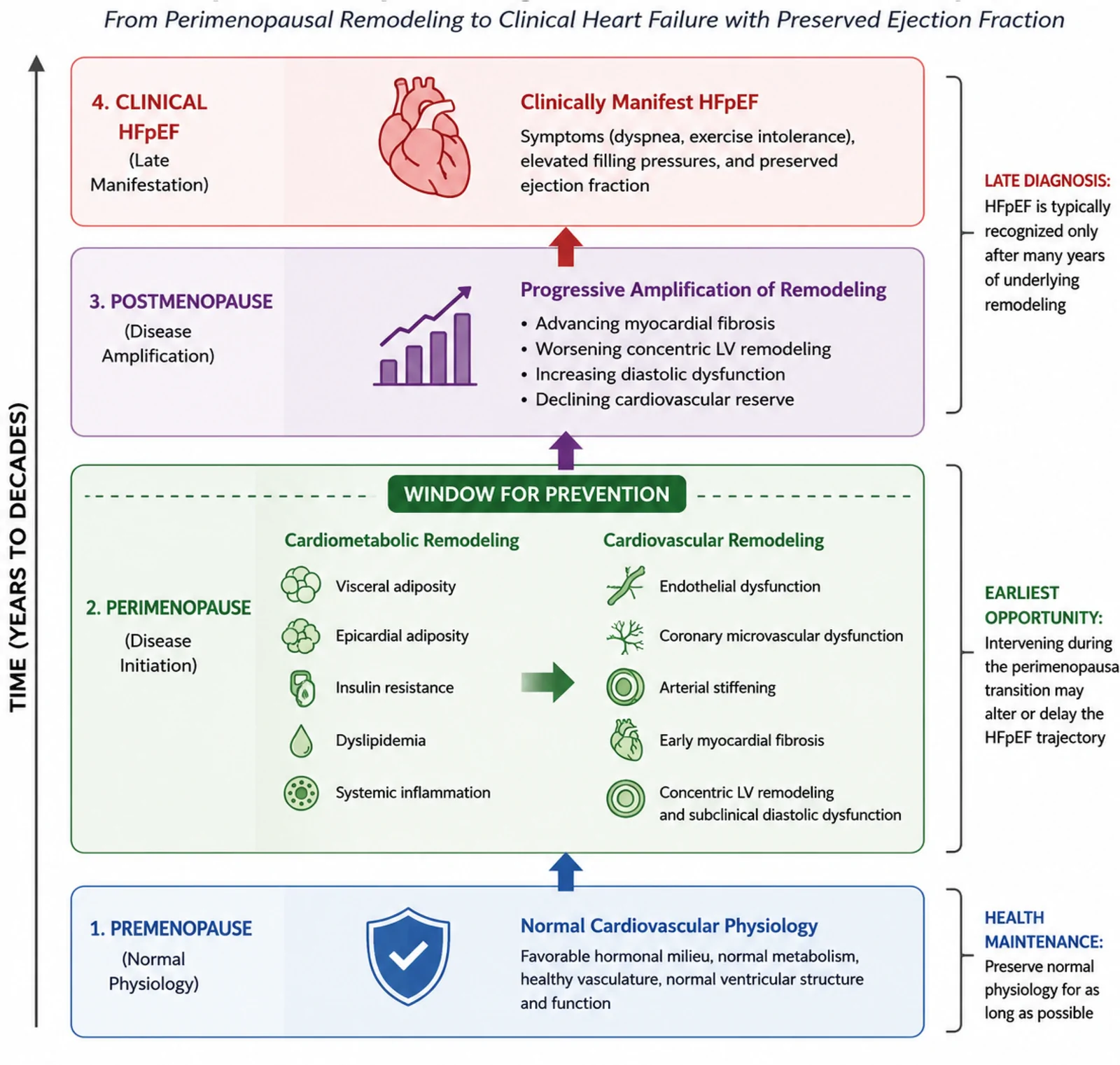
Temporal origin framework of female-predominant heart failure with preserved ejection fraction: From perimenopausal remodeling to clinical manifestation.

Within the proposed temporal framework, the menopausal transition represents a potential initiation or early remodeling phase in the trajectory toward female-predominant HFpEF. This proposed temporal framework is illustrated in Figure 1. During this stage, women may remain clinically asymptomatic and do not fulfill diagnostic criteria for HFpEF. Nevertheless, the evidence synthesized in the previous sections suggests that several HFpEF-relevant abnormalities may already be accumulating, including visceral and epicardial adiposity, worsening metabolic function, systemic inflammation, endothelial dysfunction, vascular remodeling, and early cardiac structural changes.

The defining feature of this phase is therefore not established heart failure but the potential emergence and accumulation of its biological substrate. The abnormalities identified during perimenopause may interact across metabolic, vascular, microvascular, and myocardial compartments, progressively increasing susceptibility to subsequent disease. Importantly, perimenopause should be viewed as one component of a multifactorial susceptibility framework rather than as an independent cause of HFpEF; its effects are likely to interact with aging, hypertension, obesity, diabetes, vascular dysfunction, and other individual risk factors to determine subsequent disease development. The presence of these abnormalities should therefore not be interpreted as evidence that women already have HFpEF; rather, they may represent early remodeling that precedes the clinical syndrome.

This distinction is particularly important because the evidence remains incomplete. Direct longitudinal studies integrating reproductive staging with comprehensive assessment of CMD, myocardial fibrosis, diastolic function, and incident HFpEF are lacking. Thus, the initiation phase is inferred from the convergence of epidemiological, mechanistic, and observational evidence rather than directly demonstrated. The central hypothesis is that perimenopause may represent a period during which HFpEF-relevant remodeling begins to accumulate below the threshold of conventional clinical detection.

#### 4.6.2 Postmenopause as the amplification phase

Following the final menstrual period, the proposed disease trajectory enters an amplification phase. Rather than necessarily initiating entirely new pathological pathways, postmenopause may accelerate and consolidate abnormalities established or initiated during the menopausal transition. Persistent metabolic dysfunction, continued visceral adiposity, aging, hypertension, obesity, and other cardiovascular risk factors may interact with sustained ovarian hormone deficiency to increase vascular and myocardial burden (12).

During this phase, the biological changes previously described may become progressively more evident as structural and functional cardiac abnormalities accumulate. Ventricular remodeling, impaired relaxation, increasing myocardial stiffness, and rising filling pressures may develop or worsen as compensatory mechanisms become less able to maintain normal cardiovascular reserve (12, 39). Thus, postmenopause may represent the period during which the latent substrate assembled during perimenopause is progressively amplified rather than the point at which the disease process necessarily begins.

This distinction also provides a potential explanation for the increasing burden of HFpEF with advancing age. The menopausal transition does not occur independently of chronological ageing or conventional cardiovascular risk. Instead, the biological changes associated with reproductive aging may interact with these factors over subsequent years, accelerating remodeling in susceptible women. The proposed amplification phase therefore represents an interaction between the substrate established during the transition and the accumulating cardiometabolic burden of later life.

#### 4.6.3 Clinical HFpEF as the late manifestation

Within this temporal framework, clinical HFpEF represents the late manifestation of a pathological process that may have evolved over many years. By the time women develop exertional dyspnea, exercise intolerance, congestion, elevated filling pressures, and objective evidence of diastolic dysfunction, the cardiometabolic, vascular, microvascular, and myocardial abnormalities described above may already be well established (38, 39, 51, 52).

Clinical diagnosis should therefore be viewed as a threshold of disease recognition rather than necessarily the biological beginning of HFpEF. The proposed sequence is analogous to other chronic cardiovascular disorders in which structural and functional abnormalities develop during a prolonged preclinical period before symptoms become apparent. In female HFpEF, perimenopause may represent an earlier point on this continuum, postmenopause a period of progressive amplification, and symptomatic HFpEF the eventual clinical manifestation.

This framework does not imply that every woman who experiences perimenopausal cardiometabolic or vascular remodeling will develop HFpEF. Rather, it proposes that in susceptible women, the accumulation of these abnormalities may increase vulnerability to subsequent disease, particularly when combined with ageing, hypertension, obesity, diabetes, and other established risk factors. The clinical syndrome would therefore reflect the cumulative consequence of interacting processes rather than a single menopause-related event.

## 5 Why current research frameworks miss the earliest phase of disease

Although growing evidence suggests that important cardiometabolic changes emerge during perimenopause, this transitional stage has received relatively limited attention as a potential period of HFpEF development. This disconnect reflects not a lack of biological relevance but the predominant focus of existing studies on postmenopausal women and established cardiovascular disease. As a result, current knowledge has substantially improved our understanding of established HFpEF while offering comparatively little insight into the earliest stages of disease development.

This gap should not be interpreted as evidence that cardiovascular remodeling begins only after menopause. Instead, it reflects a broader methodological issue. Reproductive aging has rarely been incorporated into cardiovascular research as a dynamic biological process, despite increasing recognition that substantial metabolic, vascular, and myocardial changes emerge during the menopausal transition. Consequently, many studies are not structured to identify when these alterations first appear or how they evolve before symptoms develop.

### 5.1 The premenopause and postmenopause binary

A major limitation of current cardiovascular research is the continued reliance on a binary classification of women as either premenopausal or postmenopausal. Although this approach simplifies participant classification and statistical analysis, it overlooks the complex biological transition that separates these two reproductive stages. Perimenopause is neither a continuation of stable reproductive life nor simply the earliest phase of menopause. Rather, it represents a distinct period characterized by marked variability in ovarian hormone production, menstrual irregularity, and dynamic physiological adaptation (10).

This distinction has important implications for studies investigating cardiovascular risk. Women undergoing the menopausal transition are frequently grouped with premenopausal participants if menstruation persists or with postmenopausal participants once menstruation has ceased. Such categorization assumes biological similarity within each group despite considerable endocrine and metabolic heterogeneity. The consequence is not necessarily the exclusion of perimenopausal women from cardiovascular research, but their incorporation into broader categories that may obscure changes unique to the transition itself.

Consequently, the continued use of a binary reproductive framework may dilute biological signals unique to the menopausal transition and hinder efforts to define when the pathophysiological processes underlying HFpEF first emerge.

### 5.2 The timing of participant recruitment

Even when the reproductive stage is appropriately defined, the timing of participant recruitment remains an important limitation in cardiovascular research. Most observational studies and clinical trials enroll women after conventional cardiovascular risk factors or overt cardiovascular disease have already become established. Although such designs are well suited to evaluating disease progression and treatment efficacy, they provide limited insight into the biological events that precede clinical disease.

This limitation is particularly relevant to HFpEF, as many cardiometabolic alterations implicated in its pathogenesis begin to emerge during the menopausal transition. However, women are rarely recruited and followed prospectively through this period. Consequently, studies are more likely to capture the consequences of cardiovascular remodeling than its initiation, limiting the ability to determine whether interventions introduced during perimenopause could modify the trajectory toward HFpEF.

### 5.3 Limitations of existing HFpEF cohorts

Over the past two decades, dedicated HFpEF cohorts have substantially advanced understanding of the syndrome by defining its clinical characteristics, underlying mechanisms, and prognostic determinants. However, these cohorts were established primarily to investigate patients with established HFpEF rather than the biological events preceding its development. Consequently, they provide a detailed picture of the disease phenotype but limited insight into when the pathological process first begins.

This distinction is particularly important when considering the possibility that HFpEF risk may begin to emerge before clinically established disease. At the time of diagnosis, many features of HFpEF, including coronary microvascular dysfunction, myocardial fibrosis, concentric left ventricular remodeling, and impaired diastolic function, are already well established. Although these cohorts can identify mechanisms associated with disease progression and clinical outcomes, they cannot determine whether these abnormalities first emerged during the menopausal transition or evolved later in life. Addressing that question will require prospective studies that follow women before the onset of overt cardiovascular disease.

## 6 Reframing HFpEF prevention: targeting the perimenopausal window

### 6.1 The prevention window

Current approaches to HFpEF prevention are largely directed toward managing conventional cardiovascular risk factors or treating patients after structural and functional cardiac abnormalities have already developed. This strategy has improved the management of established disease but offers limited opportunity to interrupt the pathological processes that precede it. Building on prior evidence that identifies the menopausal transition as a period of accelerated cardiovascular remodeling, the temporal framework developed in this review suggests that the earliest clinically identifiable opportunity for HFpEF prevention may occur during perimenopause, when cardiometabolic remodeling first begins to accelerate.

This concept reflects a shift from viewing perimenopause solely as a reproductive transition to recognizing it as a biologically active period during which visceral adiposity, insulin resistance, endothelial dysfunction, vascular stiffening, and myocardial remodeling begin to converge. If these alterations represent the earliest stages of HFpEF development rather than isolated manifestations of reproductive aging, then preventive strategies should ideally be implemented before these changes become clinically apparent.

### 6.2 Lifestyle interventions

Lifestyle modification remains the cornerstone of cardiovascular prevention and assumes particular relevance within the temporal origin framework proposed in this review. Regular physical activity, dietary optimization, maintenance of a healthy body weight, adequate sleep, and smoking cessation target several of the mechanisms implicated in perimenopausal cardiometabolic remodeling, including visceral adiposity, impaired glucose metabolism, systemic inflammation, and endothelial dysfunction. Although these interventions are recommended throughout adulthood, their implementation and sustained adoption during the menopausal transition may provide an opportunity to attenuate the early biological changes that contribute to the subsequent development of HFpEF.

Importantly, this perspective does not imply that lifestyle interventions should be reserved for women undergoing the menopausal transition. Rather, it highlights perimenopause as a period during which existing preventive strategies may have particular biological relevance because they coincide with accelerated physiological change.

### 6.3 Menopausal hormone therapy

The temporal relationship between reproductive aging and HFpEF risk provides a biological context in which to consider menopausal hormone therapy, while not establishing a role for hormone therapy in HFpEF prevention. Current recommendations emphasize individualized prescribing based on symptom burden, age, time since menopause, and overall cardiovascular risk rather than cardiovascular disease prevention alone (53). Nevertheless, because estrogen influences endothelial function, vascular homeostasis, adipose tissue distribution, and metabolic regulation, the timing of hormone therapy may have important biological implications.

Menopausal hormone therapy has well-established effects on vascular function, metabolic regulation, and other physiological processes that are relevant to the mechanisms discussed throughout this review (10, 53). However, current evidence does not support its use solely for the prevention of HFpEF. Within the temporal origin framework proposed here, future prospective studies should examine whether initiation during the menopausal transition influences early cardiovascular remodeling, alongside its effects on long-term clinical cardiovascular outcomes.

### 6.4 Emerging cardiometabolic therapies

The emergence of therapies with broad cardiometabolic benefits has created additional opportunities to reconsider prevention across the female life course. Sodium glucose cotransporter 2 inhibitors have demonstrated benefits in heart failure populations, including patients with preserved ejection fraction, while glucagon like peptide 1 receptor agonists produce substantial improvements in body weight, glycemic control, and multiple cardiometabolic risk factors (54). Both therapeutic classes target pathways that overlap with mechanisms implicated in perimenopausal remodeling.

At present, however, these agents are used to treat established metabolic disease or clinically recognized heart failure rather than to prevent HFpEF during the menopausal transition. Whether earlier initiation in appropriately selected women could modify the trajectory from cardiometabolic remodeling to overt HFpEF remains unknown and represents an important question for future prospective investigation.

### 6.5 Toward a sex-informed prevention paradigm

Collectively, these considerations support a broader shift toward a sex-informed approach to cardiovascular prevention that explicitly incorporates reproductive aging into risk assessment. Rather than viewing menopause simply as a demographic characteristic, future prevention strategies may benefit from recognizing the menopausal transition as a period of heightened biological vulnerability during which cardiometabolic and cardiovascular remodeling accelerates.

If this temporal relationship is confirmed in longitudinal studies, the menopausal transition could represent an important window for earlier cardiovascular risk identification and prevention of HFpEF in women. This approach would involve identifying women who develop adverse cardiometabolic or cardiac changes during perimenopause and determining whether targeted interventions at this stage can modify subsequent disease trajectories. Such a paradigm would shift prevention upstream, from management of clinically established HFpEF toward earlier identification and modification of potentially modifiable risk factors before more advanced structural and functional cardiac abnormalities develop.

## 7 Future directions

### 7.1 Longitudinal cohorts

A major priority for future research is the establishment of longitudinal cohorts that recruit women before or during the menopausal transition and follow them prospectively into later life. Such cohorts would provide a unique opportunity to characterize the temporal evolution of cardiometabolic and cardiovascular remodeling across reproductive aging rather than relying on retrospective reconstruction after disease has become clinically apparent. Standardized reproductive staging using the STRAW+10 framework, combined with serial assessment of cardiometabolic risk factors and cardiovascular function, would enable a more precise understanding of when early remodeling emerges and how these changes relate to subsequent cardiovascular outcomes, including HFpEF.

However, an important methodological challenge is that reproductive stage is often determined retrospectively, particularly when definitive staging depends on subsequent menstrual patterns. This limits the ability to align biomarkers and cardiovascular phenotypes precisely with the stage in which they emerge and complicates assessment of whether stage-specific changes predict subsequent HFpEF risk. This limitation should be recognized when interpreting longitudinal studies of reproductive aging and cardiovascular risk.

### 7.2 Deep cardiovascular phenotyping

Longitudinal cohorts should be complemented by comprehensive cardiovascular phenotyping capable of detecting subclinical disease before the onset of symptoms. Repeated assessment of coronary microvascular function, endothelial function, arterial stiffness, cardiac structure, myocardial tissue characteristics, and diastolic performance may help define the sequence through which physiological adaptation progresses to overt cardiovascular remodeling. Integrating these measurements with reproductive staging would provide a more complete understanding of disease evolution across the menopausal transition.

### 7.3 Biomarker discovery

The identification of biomarkers that reflect early cardiometabolic and myocardial remodeling represents another important research priority. Circulating markers of inflammation, fibrosis, endothelial dysfunction, and metabolic dysregulation, together with emerging multiomics approaches, may facilitate earlier detection of women at greatest risk while providing mechanistic insight into the biological pathways linking perimenopause with HFpEF.

### 7.4 Prevention trials

Future intervention studies should move beyond treating established HFpEF and evaluate whether preventive strategies introduced during perimenopause can modify the trajectory of cardiovascular remodeling. Such trials may examine optimized lifestyle interventions as well as selected pharmacological therapies in appropriately defined populations, with emphasis on mechanistic cardiovascular endpoints alongside conventional clinical outcomes.

### 7.5 Future evaluation of the temporal origin framework

Ultimately, the temporal origin framework should be evaluated through the integration of longitudinal clinical data, advanced cardiovascular phenotyping, biomarker profiling, and prevention trials. Confirmation that perimenopausal cardiometabolic remodeling consistently precedes and predicts subsequent HFpEF would support a fundamental shift in current concepts of disease development. Conversely, failure to demonstrate these temporal relationships would refine or challenge the proposed model. Either outcome would advance understanding of the origins of HFpEF and inform more effective, sex-specific approaches to cardiovascular prevention.

## 8 Conclusion

Heart failure with preserved ejection fraction has emerged as the predominant form of heart failure in women, yet the biological events that precede its clinical development remain incompletely understood. Contemporary research has substantially improved understanding of the syndrome once structural and functional cardiac abnormalities are established, but considerably less attention has been directed toward identifying the earlier stages at which these pathological processes may emerge. This review highlights the menopausal transition as a potentially relevant period for further investigation in this context.

The evidence synthesized throughout this review suggests that perimenopause is accompanied by changes in body fat distribution, metabolic regulation, vascular function, systemic inflammation, and cardiovascular structure that overlap with biological mechanisms implicated in HFpEF. However, direct longitudinal evidence linking these changes to subsequent HFpEF remains limited. The available epidemiological, mechanistic, observational, and preclinical evidence, therefore, supports biological plausibility but does not establish that perimenopausal remodeling independently initiates or causes HFpEF. Rather, these changes may contribute to cardiovascular susceptibility in the context of aging, hypertension, obesity, diabetes, vascular dysfunction, and other individual risk factors.

The temporal framework presented in this review provides a conceptual means of organizing these observations across the female life course, from potential perimenopausal remodeling through postmenopausal amplification to clinical HFpEF. Importantly, this framework should be regarded as a testable conceptual model rather than an established causal disease trajectory. Whether the timing and magnitude of perimenopausal remodeling predict subsequent structural cardiac abnormalities, diastolic dysfunction, or incident HFpEF remains to be determined.

Ultimately, the value of this framework lies in the research questions it generates. Prospective longitudinal cohorts incorporating standardized reproductive staging, comprehensive cardiovascular phenotyping, biomarker profiling, and appropriately designed prevention studies will be essential to determine whether cardiovascular changes observed during the menopausal transition predict subsequent HFpEF and to clarify their contribution to disease development. Clarifying when clinically relevant HFpEF-related remodeling emerges may prove as important as understanding why it develops. Recognizing perimenopause as a potentially critical window of cardiovascular remodeling could therefore open new opportunities for earlier risk stratification, targeted intervention, and more effective prevention of HFpEF in women, if this temporal relationship is confirmed by future longitudinal evidence.
